# Prenatal Diagnosis of a Ductal-Dependent Branch Pulmonary Artery: Extra Vessels in the 3-Vessel and Trachea View

**DOI:** 10.3390/jcdd11020055

**Published:** 2024-02-04

**Authors:** Anmol Goyal, Maria Kiaffas, Tara Swanson, Melanie J. Kathol, Sanket Shah, Nitin Madan

**Affiliations:** 1 Ward Family Heart Center, Children’s Mercy Hospital, Kansas City, MO 64108, USA; 2 Department of Pediatrics, University of Missouri-Kansas City, Kansas City, MO 64108, USA; 3 Kansas City Pediatric Cardiology, Kansas City, MO 64116, USA

**Keywords:** bilateral ductus, anomalous pulmonary artery, prenatal diagnosis, 3-vessel view, fetal echocardiography

## Abstract

Obtaining a three-vessel (3V) and three-vessel and trachea (3VT) view from the fetal upper mediastinum is now considered to be part of standard imaging protocol for routine obstetric cardiac screening examinations. We report two fetal cases of an anomalous pulmonary artery origin, utilizing the standard 3V and 3VT views. Further imaging led to a rare diagnosis of bilateral ductus arteriosus with discontinuous branch pulmonary arteries in the absence of any other congenital heart defect. We briefly discuss the imaging features, differential diagnoses, and management of this rare entity.

## 1. Introduction

Accurate prenatal diagnosis of congenital heart defects (CHDs) has been associated with improved outcomes, especially in ductal dependent lesions [1,2]. The importance of the three-vessel (3V) view in the fetal diagnosis of ventricular outflow tract and/or great artery anomalies was introduced by Yoo et al., 1997 [3]. Since then, multiple studies have demonstrated its utility in identifying conotruncal abnormalities such as transposition of the great arteries (TGA), tetralogy of Fallot (TOF), TOF with pulmonary atresia, and truncus arteriosus. Additionally, lesions such as coarctation of the aorta, right aortic arch, and vascular rings can also be diagnosed using this view [4]. Recognizing their importance, The International Society of Ultrasound in Obstetrics and Gynecology included the 3V and three-vessel and trachea (3VT) view as part of routine cardiac screening examination in 2013 [5].

We report two fetal cases of anomalous branch pulmonary artery (PA) origin suspected due to abnormal 3V and 3VT views. Further imaging was suggestive of bilateral ductus arteriosus (DA) with discontinuous branch PAs.

Bilateral DA is an uncommon abnormality usually associated with pulmonary atresia, aortic arch anomalies, and heterotaxy syndromes with complex CHD [6,7]. Bilateral DA with discontinuous branch PAs without associated CHD is a rare entity. Prenatal diagnosis is crucial for preventing development of postnatal branch PA isolation following ductal closure and subsequent development of pulmonary vascular disease in the lung fed from the pulmonary trunk [8].

## 2. Cases

### 2.1. Case 1

**Figure 1:**
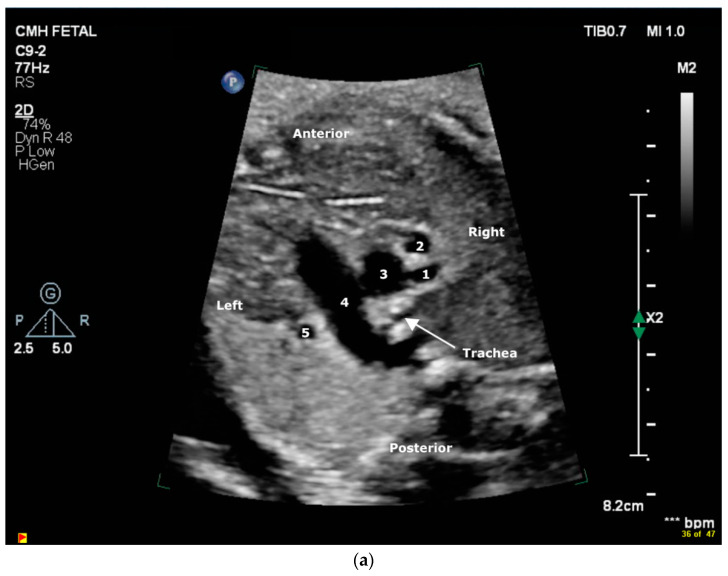

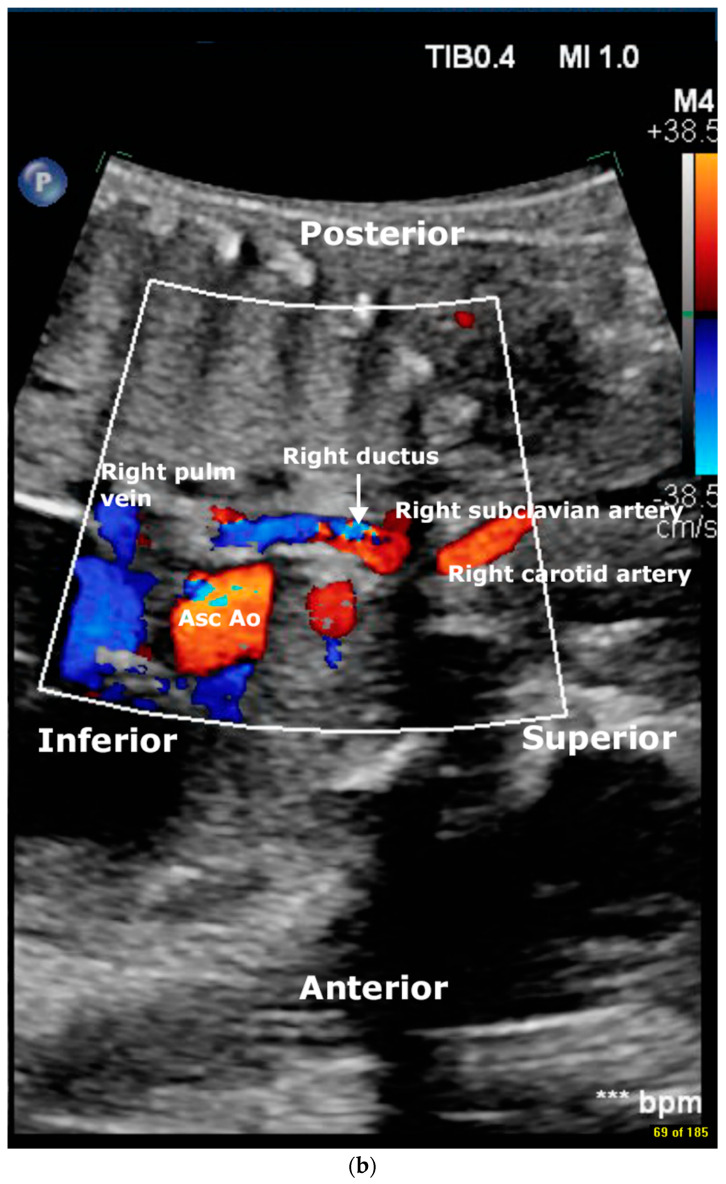

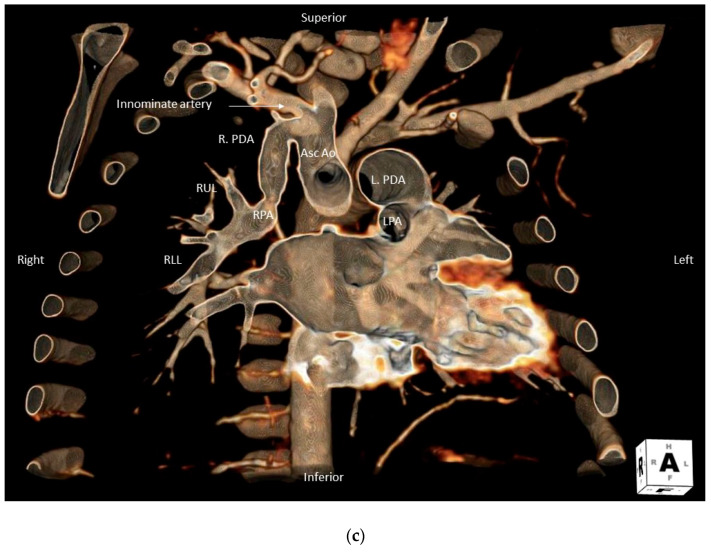
(**a**) Abnormal “5-vessels” noted in a standard 3VT fetal echocardiography view. Viewed from right to left, these vessels include right pulmonary artery (1), right superior vena cava (2), aorta (3), main pulmonary artery (4), and left superior vena cava (5). (**b**) Fetal echocardiogram sagittal plane color Doppler image showing the anomalous origin of the right pulmonary artery (RPA) from an aortic arch branch. Asc Ao, Ascending aorta; Right pulm vein, right pulmonary vein. (**c**) Computed tomography angiography (CTA) coronal view showing the right pulmonary artery origin from a right ductus arteriosus from the base of the innominate artery. R. PDA, right-sided patent ductus arteriosus; RPA, right pulmonary artery; RUL, right upper lobe branch; RLL, right lower lobe branch; Asc Ao, ascending aorta; L. PDA, left-sided patent ductus arteriosus; LPA, left pulmonary artery.

A 21-year-old woman was referred to our center for fetal echocardiography at 28-week gestation for suspected TGA. The initial study demonstrated normal cardiac segmental anatomy with normal biventricular size and systolic function. A persistent left superior vena cava (LSVC) to coronary sinus was present. The aortic arch was left-sided with borderline small dimensions of the transverse arch and diastolic flow reversal. The right pulmonary artery (RPA) was sub-optimally imaged. Due to concerns of coarctation and difficulty in delineating the RPA, a follow-up fetal echocardiogram was performed at 32-week gestation, which revealed the presence of 5 vessels in the 3V and 3VT views (Figure 1a). There was an absence of main pulmonary artery (MPA) to RPA continuity. The vessel presumed to be the RPA had a vascular connection (was fed or was perfused by a branch of) to the aortic arch (Figure 1b) and presented as a fifth vessel in addition to the LSVC. The origin of the RPA was suspected to be from either a right-sided DA or an aortopulmonary collateral (APC). The aortic isthmus was trivially hypoplastic, and diastolic flow reversal was attributed to probable run-off due to the abnormal RPA origin. The family was counseled regarding delivery at a tertiary care center with the possible need for postnatal Prostaglandin E1 (PGE1) infusion and neonatal intervention.

A full-term female weighing 3 kg was born by spontaneous vaginal delivery. Initially stable on room air, the newborn developed intermittent desaturations to the 70–75% range while awaiting a postnatal echocardiogram. The echocardiogram confirmed the discontinuity of the RPA from the MPA. The RPA origin appeared to be from the base of the innominate artery, but the vasculature in this region appeared much smaller than illustrated on fetal imaging. This prompted concerns about the RPA’s blood supply originating from a right-sided DA rather than an APC. Prompt initiation of PGE1 infusion was undertaken to improve RPA flow and prevent RPA isolation, and subsequent gated computed tomography angiography (CTA) verified the origin of the RPA from a right-sided DA which was originating from the base of the innominate artery (Figure 1c). Right DA and RPA diameters increased substantially compared to initial postnatal echocardiogram. Concurrently, oxygen saturations improved, likely related to the resolution of some newborn respiratory distress syndrome and/or improvement in ventilation/perfusion mismatch, which could have resulted from ductal constriction and reduced perfusion to the right lung. Genetic testing was negative for 22q11 deletion syndrome. The neonate underwent successful surgical reimplantation of RPA on day of life 12.

### 2.2. Case 2

A 22-year-old woman underwent a routine anatomy scan at 20-week gestation and concerns were raised regarding an abnormality of the aortic valve, in presence of normal biventricular size and systolic function. A follow-up echocardiogram was performed, raising concerns for possible coarctation. The pulmonary artery anatomy was not well delineated. The family was counselled regarding postnatal aortic arch monitoring and the obtaining of an echocardiogram after delivery to evaluate aortic arch and aortic valve morphology.

A full-term male was delivered via spontaneous vaginal delivery at an outside hospital and was subsequently discharged home. The newborn was asymptomatic and cleared the newborn critical congenital heart disease screening. The prenatally recommended echo was performed as an outpatient on day of life 4, and revealed normal segmental anatomy, normal biventricular size and systolic function, a right aortic arch with no coarctation, and no patent ductus arteriosus (PDA), but failed to delineate left pulmonary artery (LPA) and left-sided pulmonary veins. The patient was transferred to our center for initiation of PGE1 and further evaluation and management.

**Figure 2:**
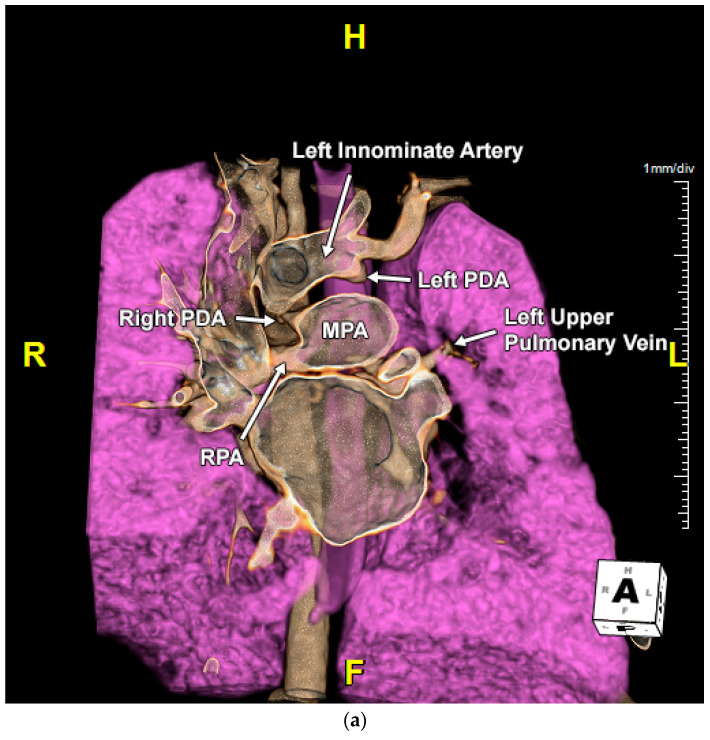

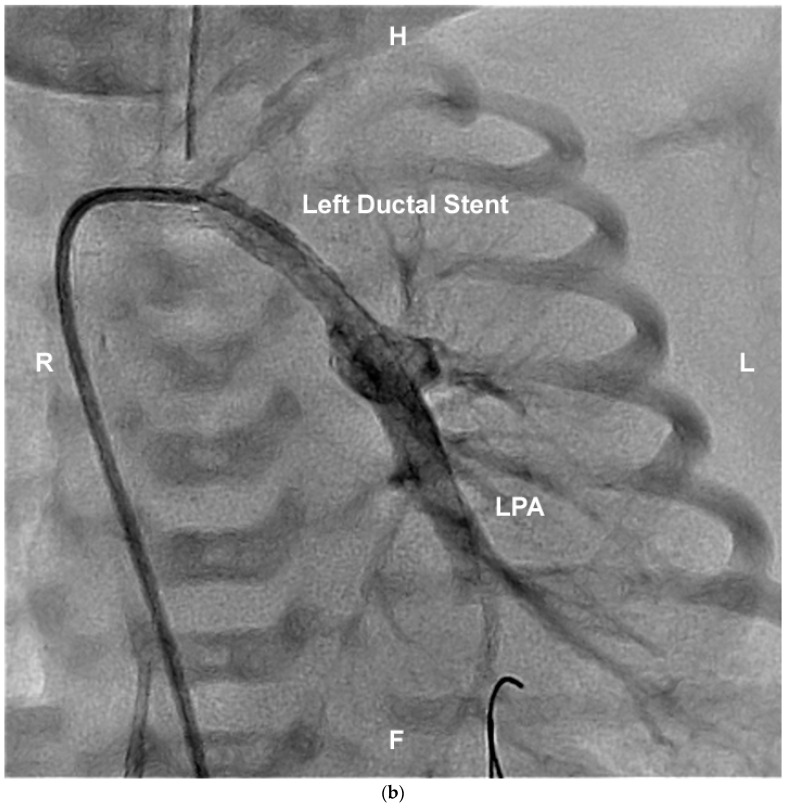

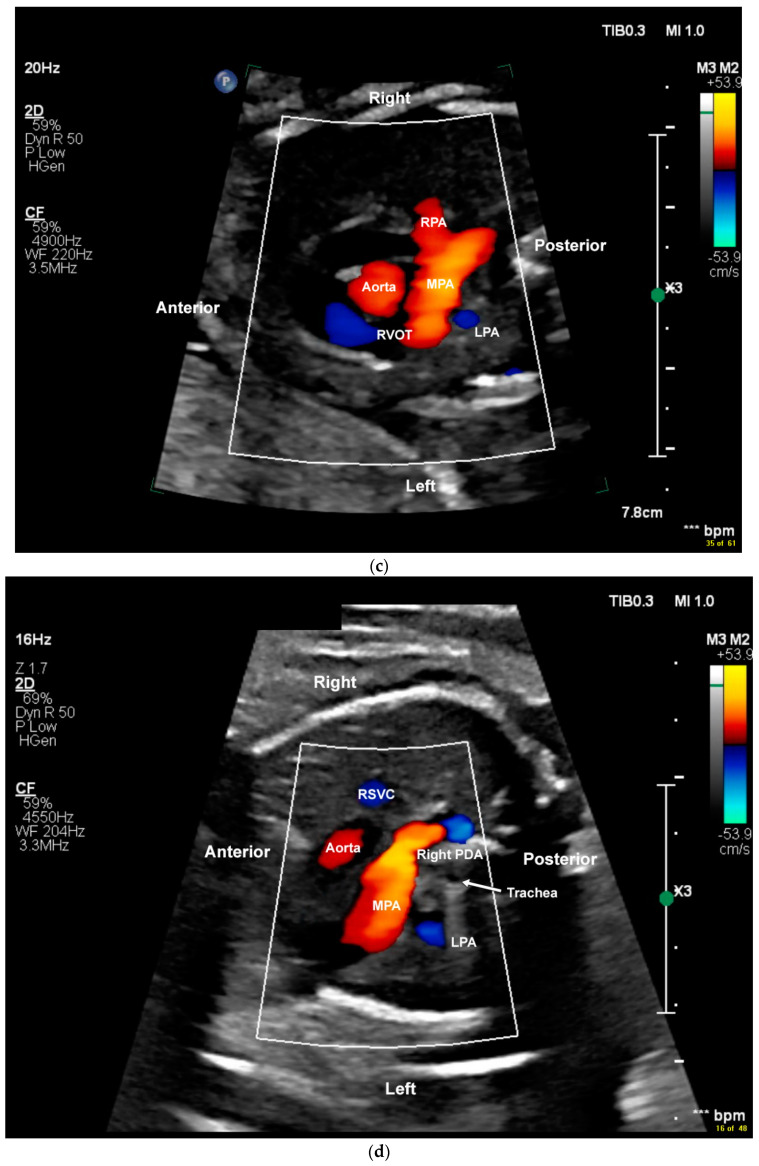

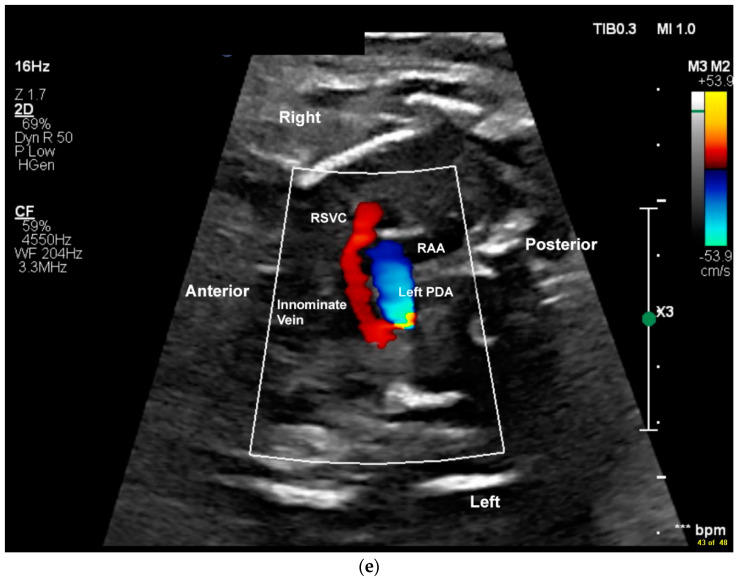
(**a**) Computed tomography angiography (CTA) coronal view showing the left ductus arteriosus dimple at the base of the left innominate artery. Right PDA, right sided patent ductus arteriosus; RPA, right pulmonary artery; MPA, main pulmonary artery; Left PDA, left-sided patent ductus arteriosus. (**b**) Anterior-posterior fluoroscopic view showing successful transcatheter ductal stenting with revascularization of the isolated left pulmonary artery (LPA). (**c**) Fetal echocardiogram color Doppler image at the level of right ventricular outflow tract (RVOT) showing the absence of bifurcation of the main pulmonary artery (MPA). LPA, left pulmonary artery; RPA, right pulmonary artery. (**d**) Abnormal 3-vessel and trachea (3VT) view with color Doppler. Viewed from right to left, these vessels include right superior vena cava (RSVC), aorta, main pulmonary artery (MPA) and left pulmonary artery (LPA). Right PDA, right-sided patent ductus arteriosus. (**e**) Fetal echocardiogram color Doppler image superior to the 3-vessel and trachea (3VT) view showing the left patent ductus arteriosus (PDA) origin and its course towards the left lung. This image also shows a right-sided aortic arch (RAA). RSVC, right superior vena cava.

Repeat echocardiogram at our facility demonstrated a right-sided DA with no evidence of a left-sided DA. The LPA could not be identified. However, left-sided pulmonary veins could be profiled by 2D imaging, but there was difficulty demonstrating flow by color Doppler. A gated CTA showed normal left-sided pulmonary venous anatomy but no LPA. A ductal dimple was appreciated underneath the left innominate artery suggestive of bilateral DA with LPA isolation (Figure 2a). Genetic testing was negative for 22q11 deletion syndrome. After a multi-disciplinary discussion, the patient was taken to the cardiac catheterization lab and underwent a successful ductal stenting with revascularization of the isolated LPA (Figure 2b).

## 3. Discussion

The presence of bilateral DA in the absence of intracardiac defects is extremely rare, and is often accompanied by an anomaly of the great arteries. Its prenatal diagnosis is challenging. Careful attention to the 3V and 3VT views can identify abnormalities in ventriculoarterial alignments, number, and arrangement of the great vessels, and provide clues to the correct diagnosis. In our first case, in addition to the readily recognizable LSVC, the absence of MPA bifurcation and the presence of a fifth vessel (presumed to be the RPA) led to further interrogation of the vessel’s course and its reverse tracing from the hilum to its origin. This effort identified the vessel’s origin from the base of the innominate artery, ruling out the diagnosis of a hemitruncus in which the RPA originates from the ascending aorta.

On retrospective review of the anatomy scan for the second case, a right aortic arch and bilateral DA with abnormal bifurcation of the MPA could be appreciated, which should have raised concerns regarding LPA isolation following postnatal PDA closure (Figure 2c–e).

**Figure 3:**
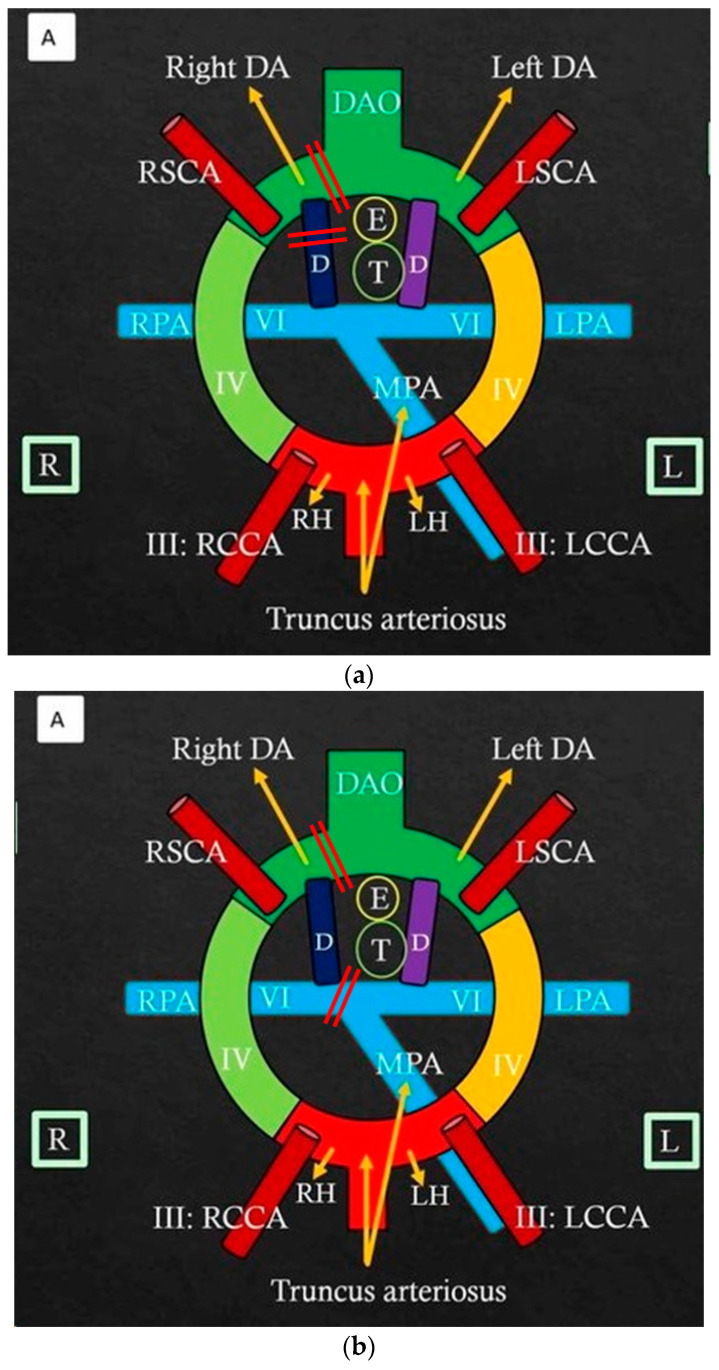

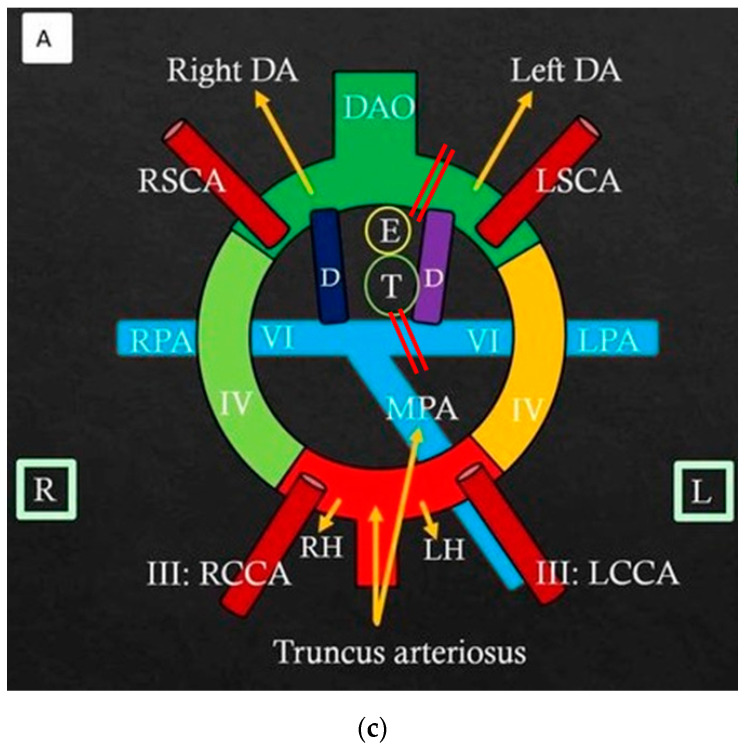
(**a**) Edwards hypothetical paired aortic arch model. The normal left aortic arch develops from the left horn (LH) of the aortic sac (forms the distal ascending aorta), a portion of the left 3rd pharyngeal arch artery (which also forms left common carotid artery, LCCA), the left 4th pharyngeal arch artery (which forms the distal transverse arch between LCCA and left subclavian artery, LSCA), and the left dorsal aorta. The right and left 6th pharyngeal arch gives rise to ipsilateral DA and the branch PAs, which in turn unite with the pulmonary root anteriorly. Normally, the right dorsal aorta portion and right-sided DA involute and disappear (marked by two parallel red lines) resulting in a normal left-sided aortic arch and left-sided DA. In this figure are also shown the Right subclavian Artery (RSCA), Right pulmonary artery (RPA), Right common Carotid Artery (RCCA), Right Horn (RH) of the aortic sac, Main Pulmonary Artery (MPA), and Descending Aorta (DAO). (**b**) Edwards’ hypothetical paired aortic arch model for patient # 1: The right-sided ductus arteriosus and the right pulmonary artery (RDA) persisted without connection to the main pulmonary artery (MPA). The left-sided ductus arteriosus and left pulmonary artery (LPA) connected normally to the left dorsal aorta posteriorly and MPA anteriorly. The aortic arch is left-sided, like normal, with involution of the right dorsal aorta. The two parallel red lines show the region of involution during embryological development for this patient. (**c**) Edwards’ hypothetical paired aortic arch model for patient # 2: The left-sided ductus arteriosus and the left pulmonary artery (LPA) persisted without connection to the main pulmonary artery (MPA). The right-sided ductus arteriosus and right pulmonary artery (RPA) connected normally to the right dorsal aorta posteriorly and MPA anteriorly. The left-sided dorsal aorta involuted with the resultant right-sided aortic arch. The two parallel red lines show the region of involution during embryological development for this patient. Adapted from Priya S, Atretic Double Aortic Arch: Imaging Appearance of a Rare Anomaly and Differentiation From Its Mimics.; published by Cureus, 2020 [10].

Embryologically, the truncus arteriosus gives rise to the aortic and pulmonary root, which subsequently progress to forming the aortic arch and its branches, as well as the pulmonary arterial tree. To explain the various aortic arch anomalies, Edwards has proposed a hypothetical developmental model that consists of paired aortic arches on either side, paired bilateral DA and a single dorsal aorta [9,10]. A combination of resolution and/or persistence of various structures of this model explains the anatomic variations as seen in our cases (Figure 3a–c).

An important aspect of this diagnosis was to identify the type of vessel feeding the PA, whether it was a right or left-sided DA or an APC, with the obvious implications being the need for postnatal PGE1 infusion and neonatal intervention. A DA generally arises from either the base of the innominate artery or the undersurface of the aortic arch, and follows a fairly straight mediastinal course with its pulmonary end close to the level of the MPA bifurcation. An APC may arise anywhere from the arch or descending thoracic aorta, is serpentine in course, and connects at the hilum of the pulmonary branches with the pulmonary end not visualized [6,11,12]. In both of our cases, the vessel originated from the base of the innominate artery, but its pulmonary insertion was distal to the MPA and towards the lung hilum, making an accurate differentiation between a DA and APC challenging.

Delivery planning should be altered based on the suspicion of a ductal dependent lesion in order to prevent branch PA isolation after birth. Additional testing with gated CTA complements echocardiography in delineating the entire course of the anomalous PA and is pivotal to the surgical or interventional planning, as demonstrated in our cases. To prevent pulmonary over-circulation after initiation of PGE, early surgical repair with direct anastomosis of the RPA to the MPA, using an interposition graft, was undertaken in the first case. This was similar to surgical techniques proposed for cases of hemitruncus, such as direct anastomosis or use of an interposition graft versus an MPA flap to establish connection to the pulmonary trunk [13,14,15]. In the second case, the native LPA was revascularized by stenting open the left-sided DA via a cardiac catheter-based intervention. In the future, we plan to perform a surgical anastomosis of the LPA to the MPA after allowing for some branch PA growth. Surgical management early in life usually results in an excellent anatomic and hemodynamic result [16]. Patients with an isolated unilateral absent pulmonary artery may be relatively asymptomatic. However, as they enter adolescence or adulthood, they may experience recurrent respiratory infections or develop exercise intolerance, hemoptysis, high-altitude pulmonary edema, and pulmonary hypertension in the contralateral lung [17,18,19,20]. As such, in the absence of a prenatal suspicion, this diagnosis can be completely missed, as could have happened in our second case, with potential adverse long-term consequences of losing vascular supply to a unilateral lung.

## 4. Conclusions

Accurate delineation of “extra” vascular structures in the 3V and 3VT views with the help of the above highlighted imaging tips is essential to establishing the rare fetal diagnosis of bilateral DA with discontinuous branch PAs. Prenatal identification is key to appropriate delivery planning and postnatal management.

## Data Availability

Data are contained within the article.
